# Tumour cells express functional lymphatic endothelium-specific hyaluronan receptor in vitro and in vivo: Lymphatic mimicry promotes oral oncogenesis?

**DOI:** 10.1038/s41389-021-00312-3

**Published:** 2021-03-05

**Authors:** Sini Karinen, Krista Juurikka, Roosa Hujanen, Wafa Wahbi, Elin Hadler-Olsen, Gunbjørg Svineng, Kari K. Eklund, Tuula Salo, Pirjo Åström, Abdelhakim Salem

**Affiliations:** 1 Department of Oral and Maxillofacial Diseases, Clinicum, University of Helsinki, 00014 Helsinki, Finland; 2 Cancer and Translational Medicine Research Unit, Faculty of Medicine, University of Oulu, 90014 Oulu, Finland; 3 Medical Research Centre Oulu, Oulu University Hospital and University of Oulu, 90220 Oulu, Finland; 4 Department of medical biology, Faculty of Health sciences, Arctic university of Norway, 9037 Tromsø, Norway; 5 The Public Dental Health Competence Center of Northern Norway, 9271 Tromsø, Norway; 6 Translational Immunology Research Program (TRIMM), Research Program Unit (RPU), University of Helsinki, 00014 Helsinki, Finland; 7 Department of Rheumatology, Helsinki University and Helsinki University Hospital, and Orton Orthopedic Hospital and Research Institute, 00014 Helsinki, Finland; 8 Helsinki University Hospital (HUS), 00014 Helsinki, Finland; 9 The Research Unit of Biomedicine, Faculty of Medicine, University of Oulu, 90014 Oulu, Finland

**Keywords:** Head and neck cancer, Metastasis

## Abstract

Lymphatic metastasis represents the main route of tumour cell dissemination in oral squamous cell carcinoma (OSCC). Yet, there are no FDA-approved therapeutics targeting cancer-related lymphangiogenesis to date. The lymphatic vessel endothelial hyaluronic acid receptor 1 (LYVE-1), a specific lymphatic marker, is associated with poor survival in OSCC patients. In this study, we present a potential novel mechanism of lymphatic metastasis in OSCC—lymphatic mimicry (LM), a process whereby tumour cells form cytokeratin^+^/LYVE-1^+^, but podoplanin-negative, mosaic endothelial-like vessels. LM was detected in one-third (20/57; 35.08%) of randomly selected OSCC patients. The LM-positive patients had shorter overall survival (OS) compared to LM-negative group albeit not statistically significant. Highly-metastatic tumour cells formed distinct LM structures in vitro and in vivo. Importantly, the siRNA-mediated knockdown of LYVE-1 not only impaired tumour cell migration but also blunted their capacity to form LM-vessels in vitro and reduced tumour metastasis in vivo. Together, our findings uncovered, to our knowledge, a previously unknown expression and function of LYVE-1 in OSCC, whereby tumour cells could induce LM formation and promote lymphatic metastasis. Finally, more detailed studies on LM are warranted to better define this phenomenon in the future. These studies could benefit the development of targeted therapeutics for blocking tumour-related lymphangiogenesis.

## Introduction

Oral squamous cell carcinoma (OSCC) is one of the most common cancers in the head and neck region arising anywhere in the oral cavity. Unfortunately, despite recent advances in cancer management, the overall 5-year overall survival (OS) rate remains stagnant at around 50%^1^. The poor prognosis of OSCC patients is mainly attributed to the invasiveness of OSCC cells and their ability to swiftly disseminate to regional lymph nodes^2^. Thus, there is an urgent need to better understand the mechanisms behind OSCC metastasis, and to identify novel druggable targets that can improve the survival of OSCC patients.

Vascularisation is a crucial event during tumour development and metastasis^3,4^. Intratumoural vasculature has long been thought to be formed by endothelial cells alone. However, the seminal work of Maniotis and colleagues showed that aggressive uveal melanoma cells were able to acquire endothelial cell behaviour by generating de novo vessel-like networks independently of existing vascular endothelial cells^5^. This novel paradigm, which is termed vascular mimicry (VM), has sparked an enormous interest in the field of cancer research^5,6^. Thenceforth, myriad studies have reported intriguing aspects of VM in different types of cancer (reviewed in Hendrix et al.)^6^. Interestingly, the transcriptional signature of VM-forming tumour cells revealed remarkable phenotypic plasticity (i.e. stemness), which facilitates transdifferentiation into other cell types^6,7^. Of particular importance, current antiangiogenic therapy remains ineffective on VM, thereby paving the way for more selective and personalised approaches^7^. Recently, the VM channels were shown to represent a promising prognosticator and therapeutic target in head and neck squamous cell carcinomas (HNSCC)^8^.

Lymphatic vessels in the tumour microenvironment are the main route of dissemination in carcinomas including HNSCC, where tumour cells can preferentially metastasise to several hundred of regional lymph nodes^9,10^. Furthermore, lymphatic vessel density has been shown to predict metastasis-free survival in OSCC patients better than blood microvessel density, and hence also for guiding future therapeutic approaches^11^. Mirroring angiogenesis, tumour cells were also shown to secrete lymphangiogenic factors that facilitate lymphangiogenesis and metastasis to sentinel lymph nodes^12^. Importantly, the discovery of specific markers for lymphatic endothelial cells (LEC), such as the lymphatic vessel endothelial hyaluronic acid receptor 1 (LYVE-1) and podoplanin (i.e. D2-40), has made it possible to distinguish between lymphatic and blood vessels^10,13^. Of note, LYVE‐1 bears a high degree of specificity for lymphatic vessels, and it has been an essential component of many important studies on tumour-induced lymphangiogenesis^13,14^. Moreover, LYVE-1 is strongly associated with nodal metastasis in OSCC, and its antibody was able to inhibit the development and progression of primary breast tumours^15,16^.

Based on the well-investigated concept of VM, we aimed to test our hypothesis that tumour cells can attain a LEC-like phenotype and form lymphatic vessel-like structures (i.e. lymphatic mimicry, LM) in OSCC tissue to facilitate tumour growth and metastasis. For this purpose, LYVE-1 was adopted in our study as a lymphatic marker using clinical samples, in vivo and in vitro experimental approaches. We also examined whether LM expresses other LEC markers such as D2-40.

## Results and discussion

### The CK^+^/LYVE-1^+^ vessel-like structures are identified in OSCC tumours

**Fig. 1:**
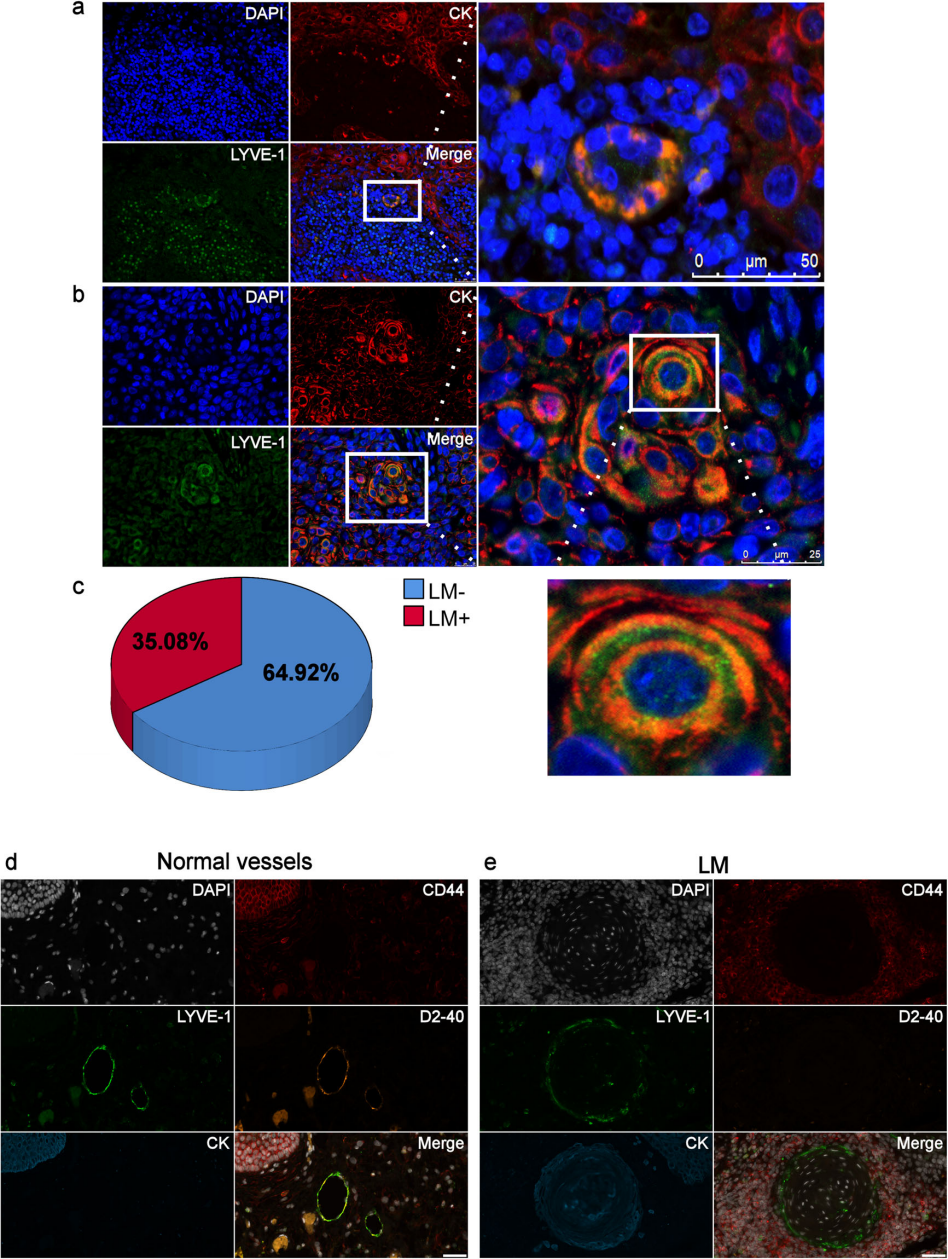
Lymphatic mimicry structures in primary oral squamous cell carcinoma (OSCC). **a** A representative figure of LM^+^ lymphatic vessel-like structure. Scale bar 50 µm. A total of 57 OSCC patient primary tumour samples were stained with CK/LYVE-1 and scored as LM^+^ or LM^−^ based on the criteria detailed in the results and discussion section. The paraffin-embedded resection specimens were obtained from patients with OSCC of the mobile tongue. Patients were treated surgically at Oulu University Hospital during the period of 2009–2016 with no other preoperative treatment. Informed consent was obtained from all patients. The use of these samples and data enquiry were approved by the Ethics Committee of the Oulu University Hospital and by the National Supervisory Authority for Welfare and Health (VALVIRA). For the double-label immunofluorescence (IF) staining, deparaffinised sections of human (*n* = 57) and mouse (*n* = 18) OSCC tissues were pretreated with 10 mM Citrate buffer (pH 6) and underwent microwave antigen retrieval in a Micromed T/T Mega Microwave Processing Lab Station (Hacker Instruments & Industries). Blocking of non-specific binding was performed in 10% donkey normal serum (Sigma-Aldrich) for 1 h at room temperature (RT). Blocking of non-specific binding was performed in 10% donkey normal serum (Sigma-Aldrich) for 1 h at room temperature (RT). Slides were then incubated with an antibody solution containing 1 µg/ml polyclonal rabbit antihuman LYVE-1 antibody (HPA042953, Sigma‐Aldrich, St Louis, MO, USA), and 1:200 diluted monoclonal mouse antihuman pan-cytokeratin (CK; DAKO, Carpinteria, CA, USA) overnight at +4 °C. Human lymph node tissue was utilised as a positive control for LYVE-1. For negative controls, the primary antibodies were omitted or replaced with an antibody isotype control. After washing 3x in phosphate buffered saline (PBS), slides were then incubated with: (1) donkey anti-mouse Alexa Fluor^®^‐568 and donkey anti-rabbit Alexa Fluor^®^‐488 conjugated secondary antibodies (1:200; Vector Laboratories) for 1 h at RT and washed in PBS; (2) 4',6-Diamidino‐2‐phenylindole (DAPI; 1:2000; Sigma-Aldrich, Germany) for 10 min at RT and washed 2 times in PBS and once in dH2O. Imaging was performed using the fully automated Leica DM6000 microscope supplied with the Leica DF365-FX camera (Leica Microsystems, Wetzlar, Germany). **b** A representative figure of LM^+^ cluster with an insert showing that LYVE-1 immunoreactivity is detected in the tumour cell membrane and cytoplasm. Scale bar 25 µm. **c** The distribution of LM^+^ and LM^−^ in the clinical samples from randomly selected OSCC patients (*n* = 57). **d** A representative figure of normal lymphatic vessels in OSCC sample mapped with different LM-relevant markers using the multiplexed immunohistochemistry (mIHC). The results revealed that normal vessels were expressing both LYVE-1 and D2-40 with a faint expression of CD44. **e** A representative figure of LM^+^ OSCC sample mapped by the same mIHC panel. The results revealed a strong immunoexpression of CK^+^/LYVE-1^+^/CD31^+^ but completely D2-40-negative in these vessel-like structures. Scale bar 50 µm. The following antibodies were included in the mIHC panel: LYVE-1 (as above); 1:100 diluted monoclonal mouse antihuman D2-40 (Dako, Carpinteria, CA, USA); 1:100 diluted monoclonal mouse antihuman CD44 (Cell Marque, Rocklin, CA, USA); and a mix of two CK clones: (1) 1:150 diluted C-11 (Abcam, Cambridge, UK), (2) 1:100 diluted AE1/AE3 (Dako, Carpinteria, CA, USA). The mIHC was conducted on randomly selected OSCC samples (*n* = 5) from the LM^+^ group. The mIHC was carried out in the Digital Microscopy and Molecular Pathology Unit (FIMM institute, University of Helsinki) as described in Blom et al.^47^.

First, we examined the presence of lymphatic vessel-like structures (i.e. LM) in primary OSCC tumours (*n* = 57) using specific tumour and LEC markers (CK and LYVE-1, respectively). The following criteria were set to identify the LM phenomenon in tumour tissues: (1) intratumoural vessel- or capillary-like structures; (2) LM lining is positive for OSCC tumour marker (CK^+^) staining; and (3) positive for LEC marker (LYVE-1^+^) staining. Interestingly, the OSCC tissues contained vessel-like structures lined by CK^+^/LYVE-1^+^ cells as depicted in (Fig. 1a**)**. In addition, some CK^+^/LYVE-1^+^ cells were also seen as a few “hot spots” in nests of densely packed tumour cells, where LYVE-1 immunoreactivity was observed in the tumour cell membrane and cytoplasm (Fig. 1b). These LM structures were observed in 20/57 (35.08%) OSCC patients (Fig. 1c). We then assessed whether LM structures express other LEC markers such as D2-40, which represents, together with LYVE-1, the most commonly used LEC markers in HNSCC^10^. Also, we tested the status of CD44, which enhances tumour aggressiveness by promoting tumour cell plasticity and VM^17^. Using the multiplexed immunohistochemistry (mIHC), normal lymphatic vessels were LYVE-1^+^/D2-40^+^ (Fig. 1d). However, LM structures were strongly CK^+^/LYVE-1^+^/CD44^+^ but entirely lacking D2-40 immunoreactivity (Fig. 1e).

### The prognostic value of LM status in OSCC patients

To investigate the clinical relevance of LM expression with the survival and the clinicopathological parameters of OSCC patients, we divided the patients (*n* = 57) into two groups based on the LM status: positive (LM^+^) and negative (LM^−^) groups. Interestingly, the estimated OS of the LM^+^ group (58 months) was noticeably shorter compared to the LM^−^ group (80 months) (Supplementary Fig. 1). Likely due to a small sample size, such difference did not however reach a statistical significance (*P* = 0.351). Yet, the correlation between LM^+^ status with younger age group (*P* = 0.016) and higher clinical T-stage (*P* = 0.077) represented interesting hints (Supplementary Table 1). The investigators were blinded to the clinical data of the patients during the experiment and when assessing the outcome by using samples with coded labels, with no reference to any respective group.

### Tumour cell lines express LYVE-1

**Fig. 2:**
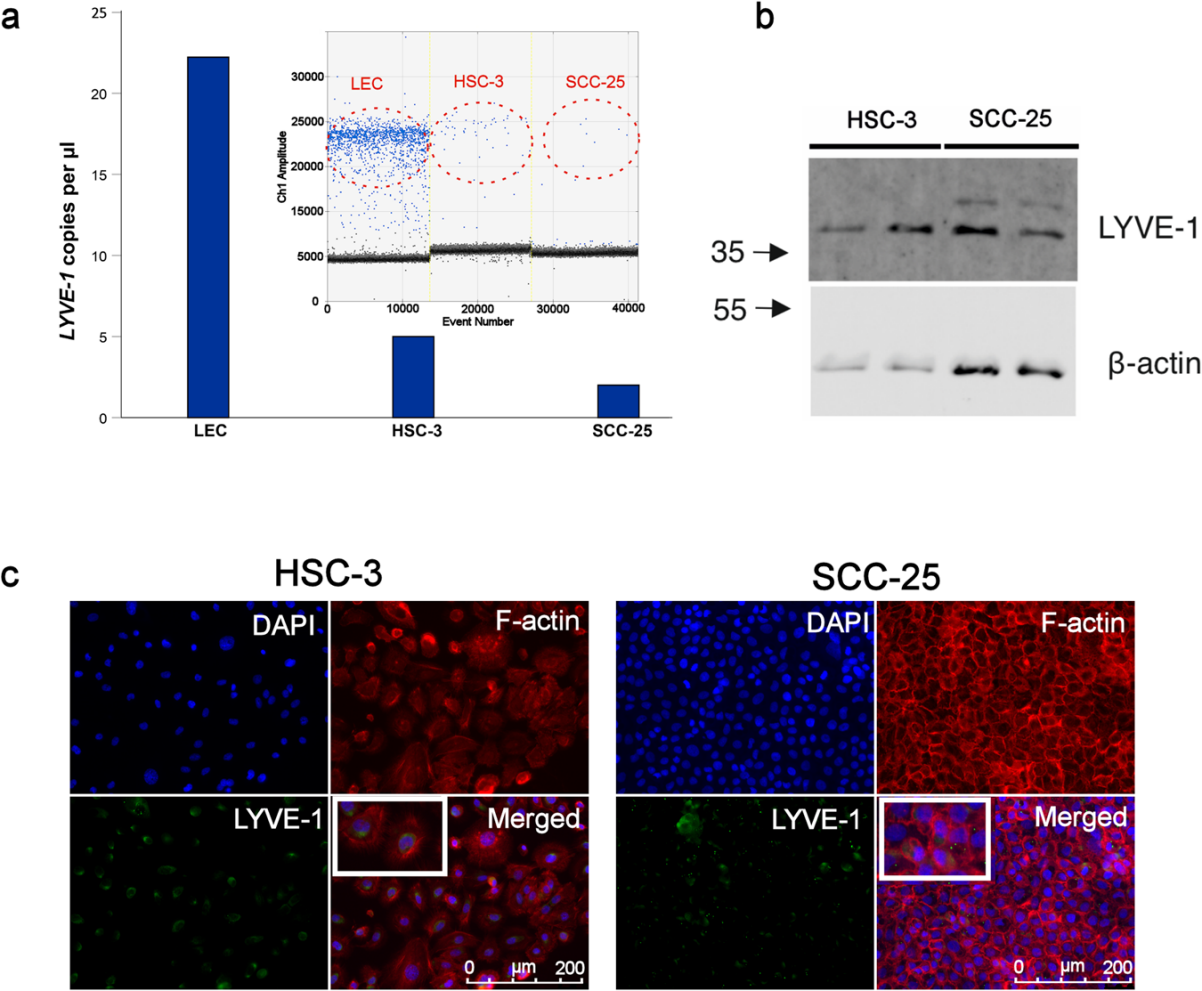
OSCC cell lines express the lymphatic endothelium-specific LYVE-1. **a** Droplet-digital PCR analysis revealed a positive expression of LYVE-1 in OSCC cell lines. The used cell lines are detailed in the supplementary materials. The ddPCR was performed as recently reported^48^. The LYVE-1 primers were designed using the Primer-BLAST tool (powered by Primer3) in NCBI website. The primers were checked by BLAST analysis to avoid any non-specific binding. The LYVE-1 primer sequences were as follows: forward 5′-GTGAGCAAAAAGGCGAACC-3′; reverse 5′-AATCCATCTCCAACCCAGC-3′. Detection of positive droplets and data analysis were performed using QX200^™^ Droplet Digital^™^ PCR Systems (Bio-Rad Laboratories) and QuantaSoft software (version 1.7.4.0917; Bio-Rad Laboratories) according to the manufacturer’s instructions. **b** The LYVE-1 protein was also detected in both OSCC cell lines by immunoblotting. The immunoblotting details can be found in the supplementary materials. **c** Double IF staining revealed positive LYVE-1 immunoreactivity in both OSCC cell lines. HSC-3 and SCC-25 cells were cultured on coverslips, washed in PBS and fixed using 4% paraformaldehyde. Cells were then stained with the same LYVE-1 antibody as above, and incubated with Alexa Fluor^™^-568 Phalloidin for filamentous actin (F-actin; 1:200; Thermo Fisher Scientific) for 30 min at RT. Next, cells were stained with DAPI (1:2000) and mounted with ProLong^®^ Gold Antifade Mountant (Thermo Fisher Scientific). Scale bar 200 µm.

To confirm that the detected intratumoural LYVE-1-immunoreactivity was derived from tumour cells and not from other cells (e.g. vicinal LEC), we first quantified LYVE-1 mRNA in OSCC cells using the highly sensitive droplet-digital PCR (ddPCR). The ddPCR reactions with at least 13 × 10^3^ droplets per sample were accepted for further analysis. The absolute quantification analysis revealed that OSCC cells express LYVE-1 gene. LYVE-1 copies were detected in the cells with high-metastatic potential (i.e. HSC-3; 5 copies/μl) and also in the low-metastatic SCC-25 cells (2 copies/μl) (Fig. 2a). LYVE-1 protein was also detected in both OSCC cell lines by immunoblotting (Fig. 2b). We then utilised immunofluorescence staining to assess whether monolayered OSCC cells express LYVE-1 immunoreactivity. As expected, LYVE-1 protein was detected in the cytoplasm of both OSCC cell lines (Fig. 2c). Additionally, the expression of LYVE-1 in multiple keratinocyte and cancer cell lines was also studied (supplementary data). We found that these cell lines differentially express LYVE-1 in vitro when cultured as monolayers (Supplementary Fig. 2a, b) or in a 3D organotypic myoma model (Supplementary Fig. 2c).

### The high-metastatic HSC-3 cells form LYVE-1+ vessel-like structures in vitro and in vivo

**Fig. 3:**
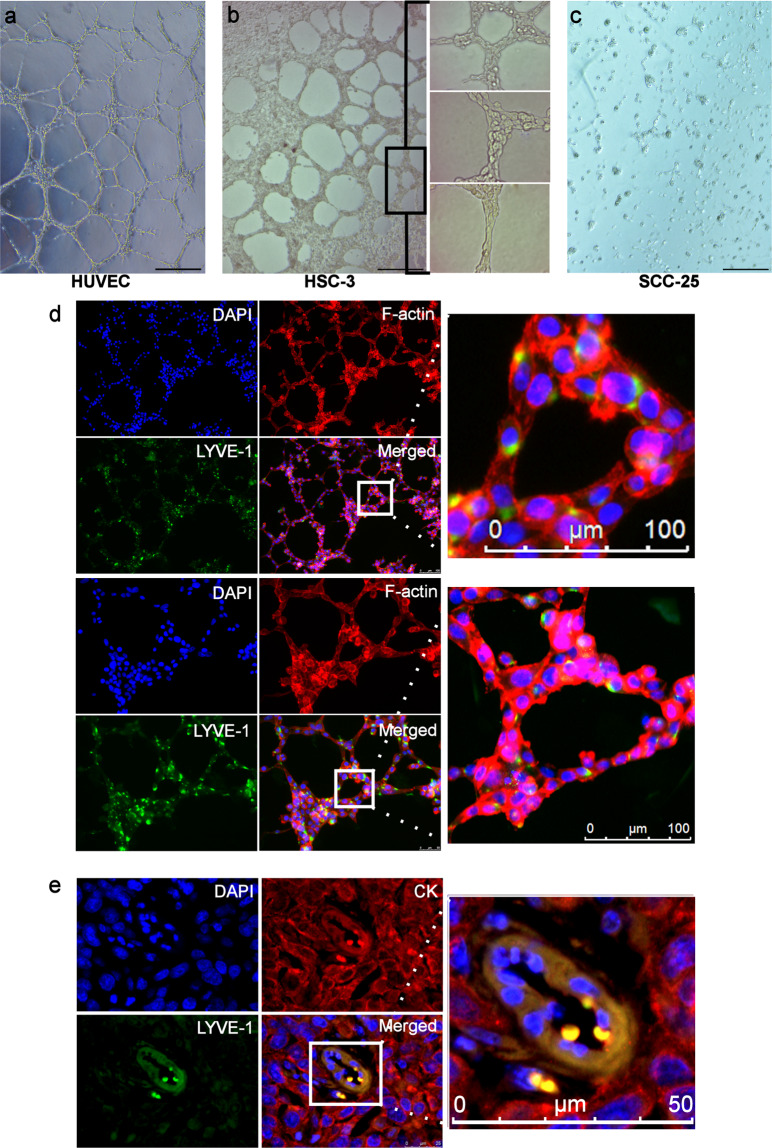
The high-metastatic HSC-3 cells form LYVE-1^+^ vessel-like structures in vitro and in vivo. **a** Endothelial cell tubulogenesis formed by Matrigel-seeded human umbilical vein endothelial cells (HUVEC). **b** Matrigel-seeded HSC-3 cells formed well-defined, interconnected, vessel-like network that resembled the structures formed by HUVEC. **c** On contrary, the low-metastatic SCC-25 cells remained as single cells and failed to form any consistent vessel-like structures on Matrigel. Scale bar 100 µm. In brief, HUVEC and OSCC cells were first suspended in serum-free medium and seeded at a density of 20 × 10^3^ cells/well in 96-well plates pre-coated with 9.375 mg/ml Matrigel^®^ basement membrane matrix (Corning, New York, USA). Cells were then incubated for 24 h at +37 °C. **d** The HSC-3-formed vessel-like cell networks were strongly LYVE-1^+^ particularly at the tubular extensions and intercellular junctions. Scale bar 100 µm. Matrigel-seeded cells were first fixed in 4% paraformaldehyde (Santa Cruz) for 20 min at RT and washed once in PBS. Next, samples were incubated in normal serum for 1 h at RT, and the staining continued as aforementioned above. **e** The xenograft mice model of OSCC revealed formation of CK^+^/LYVE-1^+^ vessel-like structures by HSC-3 cells in the harvested tumour sections. Scale bar 50 µm. Human HSC-3 cells were detached, suspended in cold serum-free DMEM and then mixed with a cold Matrigel at 1:1 to a final concentration of 8 × 10^6^ cells/ml. BALB/c nude male mice (Charles River, Germany) were anaesthetised at age of 7 weeks via subcutaneously administered mixture of 0.5 mg ketamine and 0.1 mg xylazine hydrochloride. More details are described in the supplementary materials. Sections from 18 mice comprised primary (*n* = 9) and metastatic (*n* = 9) tumours were used for double-label IF staining as described before. The investigators were blinded to the animal group allocation during the experiment and when assessing the outcome by using samples with coded labels with no reference to any respective group. The experiments followed the ARRIVE guidelines and were conducted in accordance with the European Convention for the Protection of Vertebrate Animals for Experimental and Other Scientific Purposes´ guidelines on accommodation and care of animals. Cell culture imaging was performed using a Zeiss digital AxioCam ERc5s camera connected to Zeiss Axioplan microscope (Carl Zeiss Microscopy GmbH, Jena, Germany). Cell culture imaging was performed using a Zeiss digital AxioCam ERc5s camera connected to Zeiss Axioplan microscope (Carl Zeiss Microscopy GmbH, Jena, Germany).

After having confirmed the presence of LYVE-1 in OSCC cell lines, we examined the ability of these cells to create vessel-like structures on a 3D matrix. Interestingly, when seeded on Matrigel, the high-metastatic HSC-3 formed well-defined, interconnected, ‘vessel-like' network that resembled the early stages of endothelial cell tubulogenesis (Fig. 3a, b). In contrast, the low-metastatic SCC-25 cells failed to form any consistent structures and remained as single cells, although both cell lines were cultured under identical experimental conditions (Fig. 3c). Of note, such vessel-like cell networks were reminiscent of the LM structures in OSCC patients and exhibited stronger LYVE-1-immunoreactivity compared with the monolayer cell cultures **(**Fig. 3d**)**. To investigate the ability of HSC-3 cells to form such LM structures in vivo, cell suspension was injected into the lateral tongue border of BALB/c nude male mice (*n* = 18), followed by double-labelled IF on the harvested xenograft sections. Surprisingly, CK^+^/LYVE-1^+^ vessel-like structures, similar to those observed in patient samples, were detected in 8/18 (44.44%) of the xenograft sections, of which three were metastatic tumours (Fig. 3e).

### LYVE-1 knockdown impedes the ability of HSC-3 to form vessel-like structures on Matrigel

**Fig. 4:**
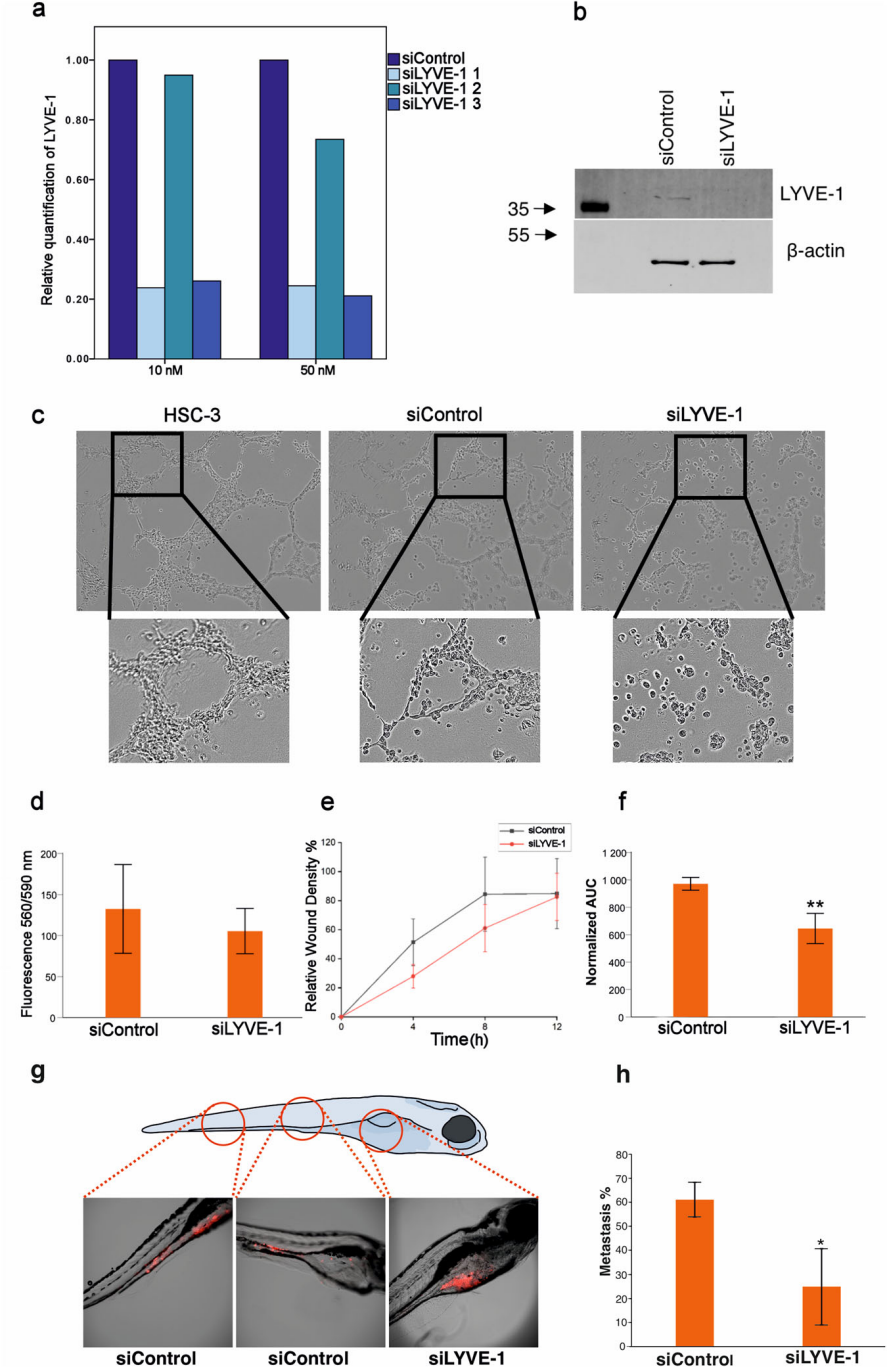
The siRNA-mediated knockdown of LYVE-1 in HSC-3 cells. **a**, **b** The qPCR and western blot analyses showed a clear attenuation of LYVE-1 in the silenced HSC-3 cells. Three Silencer Select Validated siRNAs for silencing LYVE-1 (IDs s21402, s21403 and s21404) were tested along with one negative control siRNA (cat no. 4390843, all were Ambion by Life Technologies, Carlsbad, CA, USA) in two different final concentrations, 10 and 50 nM. The siRNA sequences were as follows: (1) s21402: sense 5′-GGAUUUUGCUAUGUCAAAAtt, antisense 5′-UUUUGACAUAGCAAAAUCCaa; (2) s21403: sense 5′-CCAAAGUAGUAAAGGAGGAtt, antisense 5′-UCCUCCUUUACUACUUUGGtt; and (3) s21404: sense 5′-CAACCCUAAUGAGGAAUCAtt, antisense 5′-UGAUUCCUCAUUAGGGUUGct. For analysis of LYVE-1 gene expression, 15 × 10^4^ HSC-3 cells per well were plated on a 24-well plate in duplicates for each siRNA and concentrations. The next day the cells were transfected according to the manufacturer’s protocol with the help of Lipofectamine RNAiMAX (Invitrogen) transfection reagent. After 48 h of transfection, the RNA was extracted with TRizol Plus RNA Purification Kit (Ambion) along with manufacturer’s instructions and the RNA yield quantified with NanoDrop 2000. The LYVE-1 siRNA 1 (Lot no. AS02DZX5) at a concentration of 10 nM was determined to be optimal for functional assays. For protein extraction and functional assays, HSC-3 and SCC-25 cells (20 × 10^4^ per well) were plated on 6-well plate (Corning). The next day, LYVE-1 siRNA 1 and negative control siRNA (siControl) were transfected as described above. After 48 h, the silenced (siLYVE-1) and siControl cells were collected for protein extraction as described in the supplementary materials; or were detached, quantified and seeded for the functional experiments as described in the following sections. **c** HSC-3 cells transfected with siRNA LYVE-1 (siLYVE-1) were not able to form consistent vessel-like structures on Matrigel compared to siControl cells. The vessel-like tube formation assay was performed as described previously^49^. The siLYVE-1 and siControl cells were imaged using the IncuCyte S3^™^ live cell imaging system (Essen BioScience, Ann Arbor, MI, USA) with 10x magnification in Standard module (4 images/well) and/or 4x magnification in Whole Well module. **d** The resazurin assay revealed that siRNA-mediated knockdown of LYVE-1 caused a modest, non-significant, decrease on the growth of HSC-3 cells (*P* > 0.05). Briefly, the siLYVE-1 and siControl cells were seeded at a density of 5 × 10^3^ cells/well in a 96-well black plate (Thermo Fisher Scientific), and allowed to attach overnight. After 24 h, resazurin sodium salt (Sigma-Aldrich, Germany) was added to each well at a final concentration of 3 µg/ml, and incubated for 3 h at +37 °C. The fluorescence was measured using an excitation wavelength of 560 nm and an emission wavelength of 590 nm using a Victor 3 V instrument (PerkinElmer Life Sciences, Boston, MA, USA). **e**, **f** The IncuCyte scratch-wound healing assay showed that siLYVE-1 HSC-3 cells had a remarkable and consistent reduction in their directed migration (relative wound density) compared to the siControl HSC-3 cells. AUC area under the curve. Data were presented as means ± standard deviations. **P ≤ 0.01. The scratch-wound healing assay was performed using the IncuCyte S3^TM^ imaging system according to the manufacturer’s protocol. The siLYVE-1 and siControl cells were seeded at a density of 30 × 10^3^ cells/well in a 96-well ImageLockTM tissue culture plate and incubated for 24 h at +37 °C. Next, the WoundMaker^TM^ tool (Essen Bioscience) was used to create homogenous and consistent scratch wounds and the cells were washed once with PBS to remove dislodged cells and supplemented with 1% FBS culture media. The wells were imaged in IncuCyte S3^™^ imaging system using objective with 10x magnification in Scratch-Wound Module every 2–4 h until the open areas were closed by the tumour cells. **g**, **h** Zebrafish xenografts revealed that siControl group had more tumour metastasis to cloaca and tail areas of the fish compared with the siLYVE-1 group, where tumours remain largely confined within the yolk sac region (61.1 vs. 24.1%, respectively; *P* < 0.05). A cutoff value of ≥1 cells that metastasised outside the yolk sac region was considered to discern fish with metastasis. Data were presented as the mean of three independent experiments ± standard deviation. *P ≤ 0.05. Fish experiments were conducted at the Zebrafish Unit at the University of Helsinki and approved by the ethical permission from the regional state administrative agency (ESAVI/13139/04.10.05/2017). Wild-type zebrafish (AB strain) were bred, raised and maintained in laboratory fish multi-rack facility as described earlier^50^. Two-day post-fertilisation zebrafish larvae (*n* = 50 per experiment) were dechorionated, anesthetized with 0.04% Tricaine. Next, siControl or siLYVE-1 HSC-3 cell suspension (4 nl of 1500 cells/fish), labelled with CellTrace™ Far Red (Thermo Fisher Scientific), was microinjected into the perivitelline space of the anesthetized fish. Larvae were then maintained in an embryonic medium containing (5 mM NaCl, 0.17 mM KCl, 0.33 mM CaCl_2_ and 0.33 mM MgSO_4_; Sigma-Aldrich, Germany) at 34 °C for three days. On the fourth day post-injection, larvae were collected, fixed with 10% PFA and mounted on slides using SlowFade Gold Antifade reagent (Invitrogen, California, US). Zebrafish were imaged using Nikon Eclipse Ti-E Camera (Nikon, Tokyo, Japan) and images were analysed using ImageJ software (Wayne Rasband, NIH, US).

Given the crucial role of LYVE-1 in inducing lymphangiogenesis^18^, we next aimed to determine its putative role in the formation of LM in OSCC. To this end, we employed siRNA-mediated knockdown approach in HSC-3 cell line, while SCC-25 cells were excluded as they failed to form similar vessel-like networks. qPCR and western blot analyses showed a clear attenuation of LYVE-1 in the silenced group (Fig. 4a, b). HSC-3 cells transfected with siRNA LYVE-1 (siLYVE-1) or siControl were cultured on Matrigel for 24 h. Strikingly, siLYVE-1 treated cells showed a clear impairment in their ability to form vessel-like structures when compared to siControl cells, suggesting a pivotal role of LYVE-1 in LM formation in OSCC cells (Fig. 4c and Supplementary videos 1–3).

### LYVE-1 knockdown shows moderate, non-significant, impact on HSC-3 cell viability

To confirm that the impaired ability of siLYVE-1 cells to form vessel-like structures is not due to cell death, we utilised resazurin dye assay to assess the effect of LYVE-1 siRNA knockdown on HSC-3 cell proliferation and viability. Results revealed that transfection with LYVE-1 siRNA caused a modest, non-significant, decrease on the growth of HSC-3 cells (*P* > 0.05; Fig. 4d).

### OSCC cell migration and invasion are suppressed by LYVE-1 knockdown

It was recently shown that endogenous LYVE-1 can facilitate transluminal cell migration^19^. Thus, we assessed the influence of LYVE-1 on the tumour cell migration, a crucial metastatic feature, by employing the IncuCyte scratch-wound healing assay on siLYVE-1 and siControl OSCC cells. Notably, siLYVE-1-knocked down cells showed a remarkable and consistent reduction in their directed migration (i.e. relative wound density) compared to the siControl cells (*P* < 0.05; Fig. 4e, f). Then, we evaluated the pro-invasion potential of LYVE-1 by employing the 3D organotypic myoma model^20^. We found that siLYVE-1 cells had smaller invasion depth and invasion area compared with the siControl (Supplementary Fig. 2d–f). However, the difference was not statistically significant (*P* > 0.05).

### LYVE-1 knockdown significantly reduced metastasis in vivo

Next, we addressed the pivotal question of whether LYVE-1 is a pro-metastatic in vivo by xenotransplantation of siLYVE-1-inhibited OSCC cells into zebrafish larvae. Indeed, zebrafish is emerging as an attractive addition to animal models in cancer research, which provided important data regarding metastatic events in vivo and personalised cancer approaches^21^. The siLYVE-1 and siControl HSC-3 cells, labelled with CellTrace™ Far Red fluorochrome, were microinjected into the perivitelline space. On the fourth day post-injection, larvae were analysed by microscopic imaging to discern fish with a tumour metastasised outside the yolk sac region. Intriguingly, the siControl group had significantly more fish with metastasis to cloaca and tail regions compared with the siLYVE-1 group, where tumour remains largely confined within the yolk sac area (61.1 vs. 24.1%, respectively; *P* < 0.05) (Fig. 4g, h).

The main cause of cancer-related mortality is metastasis, a process mediated by the access of cancer cells to blood and lymphatic vasculatures^22^. Indeed, the identification of tumour cell-derived VM, as a novel model of neovascularization, has opened a new perspective in cancer research^23^. Increasing evidence shows that VM is strongly associated with poor survival in cancer patients, and hence represents an attractive therapeutic target^8,23,24^. Interestingly, in OSCC tumour tissues, we observed a phenomenon that could be similar to the VM. About one-third of randomly selected OSCC patients had vessel-like structures with surrounding CK^+^/LYVE-1^+^ cells. These structures were expressing LYVE-1 but, unlike normal LEC, they were entirely negative for D2-40, implying a key role of LYVE-1 in their development. Patients with these structures had seemingly worse survival outcome and higher clinical T-stage. However, the sample size in this report may not be large enough to reach a statistically significant difference between the LM groups. More studies with larger patient samples, across different populations, should thus be conducted to reach a resounding conclusion. Furthermore, although LYVE-1 was expressed in OSCC cell lines, only the high-metastatic cells (HSC-3) were able to form similar LYVE-1^+^ vessel-like structures in vitro and in vivo. Importantly, LYVE-1 knockdown in these cells has markedly blunted their ability to form such structures and reduced their migration, invasion and metastasis without a robust impact on cell viability. These findings may indicate a novel potential mechanism underlying tumour growth and metastasis in OSCC—LM (Supplementary Fig. 3).

Unlike sarcomas, carcinomas including HNSCC utilise lymphatic vessels as the preferential route of metastasis by initially spreading to the regional cervical lymph nodes^9,10^. Therefore, the presence of cervical lymph node metastases is considered one of the most important prognostic indicators in OSCC patients^25^. In this regard, the localisation of lymphatic vessels in the tumour tissue matters. Intratumoural LYVE-1^+^ lymphatic vessels, unlike the peritumoral ones, were indeed associated with higher relapse rate and poor disease-specific survival in HNSCC patients^26^. To our knowledge, the present study is the first to report intratumoural CK^+^/LYVE-1^+^ mosaic vessel-like structures in OSCC tumours. In support of our findings, it was recently shown that aggressive breast cancer cells can form intratumoural LM channels to access lymphatic vasculature^27^. These channels were concomitantly positive for tumour and lymphatic vessel markers including cytokeratin and LYVE-1, respectively^27^. Furthermore, we found that multiple keratinocytes and cancer cell lines differentially express LYVE-1 in vitro—a trait not widely discussed before. Interestingly, such expression levels and localisation of LYVE-1 were also retained in the 3D organotypic myoma model. The functional effects of LYVE-1 in cancer cells pose an interesting avenue for studies on cancer cell plasticity and metastatic potential.

The VM structures are traditionally identified in tumour tissues as periodic acid-Schiff positive (PAS^+^) vessel-like spaces, with the absence of any specific endothelial cell markers^28^. However, this method has been criticised for its limited specificity. In fact, PAS staining detects extracellular matrix components, and hence PAS^+^ regions may also represent non-functional structures irrelevant to VM^29,30^. On the other hand, the mosaic vessel-like pattern, where tumour and endothelial cell markers are simultaneously expressed, has gained attention not only for identifying VM but also as a prognostic marker^31,32^. This mosaic pattern was long thought to be formed by a coalescence of tumour and endothelial cells during carcinogenesis ^31^. However, recent studies revealed that aggressive tumour cells can express genes associated with non-cancerous cell phenotype, such as endothelial cell precursors^33,34^.

Phenotype plasticity allows cancer cells to reversibly transform phenotypes and acquire functional adaptation to thrive in a harsh tumour microenvironment^33,35,36^. For instance, glioblastoma cancer cells were able to differentiate into functional CD31^+^ endothelial cells, which created VM structures in a xenograft mouse model^37^. Likewise, breast cancer stem-like cells differentiated into endothelial cells and formed vessel-like structures on Matrigel^38^. Consistently with these reports, we show that LYVE-1 is expressed by OSCC cell lines, but only HSC-3 cells, and not the less aggressive SCC-25 cells, were able to form LYVE-1^+^ vessel-like network on Matrigel. The in vivo LM phenomenon was also evident in an orthotopic mouse model of OSCC. Of note, such LM vessel-like structures were strongly positive for CD44—a biomarker implicated in the plasticity of aggressive tumour cells and VM formation^17^. These findings support the notion that the mimicry phenomenon is associated with more aggressively growing tumours. A function for LYVE-1 as a lymphangiogenic factor is fully consistent with its evident expression at the tubular extensions and intercellular junctions on Matrigel. These in vitro and in vivo findings suggest that intratumoural CK^+^/LYVE-1^+^ lumens in the clinical samples are likely LM structures rather than basement membrane sleeves of pruned vessels, as could be encountered when identifying mimicry structures^8,27^.

The LYVE-1 is an essential mediator of tumour lymphangiogenesis and correlates with lymph node metastasis in HNSCC patients^18,26,39^. Indeed, LYVE-1-driven effects are mediated by binding with its ligand—hyaluronan (HA). Importantly, HA is synthesised by both tumour and stromal cells in OSCC, and it has been shown to regulate various cancer processes from initiation to metastasis^40–42^. Thus, it is logical to assume that LYVE-1^+^ OSCC cells may utilise HA/LYVE-1 interaction to facilitate oral carcinogenesis. This assumption is supported by an interesting study showing that aggressive breast cancer cells can harness their HA content to bind LYVE-1 and facilitate adhesion, hence invasion and metastasis^43^. Notably, the high-metastatic cells used in our study, HSC-3, were proven to be highly sensitive and responsive to HA-signalling in vitro^44^. Here, we have shown that HSC-3 exhibit marked suppression of tube formation, migration and invasion potential in 3D in vitro models following the siRNA-mediated knockdown of LYVE-1. More importantly, inhibition of LYVE-1 in HSC-3 cells significantly reduced their metastatic potential in zebrafish larvae. In agreement with our report, it was recently shown that anti-LYVE-1 monoclonal antibody inhibited primary tumour formation and metastasis to axillary lymph nodes in xenograft models of breast cancer^16^.

Finally, these pieces of evidence may bear considerable therapeutic implications. Despite the major advances in the development of new angiogenic inhibitors, the clinical success of these drugs remains, however, considerably limited^45^. As a result, to date there are no FDA-approved therapeutics that target tumour-related lymphangiogenesis^46^. The de novo formation of tumour-derived mimicry channels was suggested as a plausible factor that could reduce the effects of anti-angiogenic therapy^23,32^. Thus, a better understanding of LYVE-1 function in tumour cells and its role in LM formation could benefit the development of targeted therapeutics for blocking tumour lymphangiogenesis in OSCC. Clearly, more detailed studies, including larger sample size and complete deletion models of LYVE-1, are necessary in order for such phenomenon to be better defined in the future.

## Supplementary information

Supplementary materials

Supplementary Videos 1.

Supplementary Videos 2.

Supplementary Videos 3.
