# Supplementary material for: Tumour cells express functional lymphatic endothelium-specific hyaluronan receptor in vitro and in vivo: Lymphatic mimicry promotes oral oncogenesis?
Source: Oncogenesis. 2021 Mar 5;10(3):23. doi: 10.1038/s41389-021-00312-3 (PMC7977063; doi:10.1038/s41389-021-00312-3)
Supplement: Supplementary file 1 — Supplementary materials [file 41389_2021_312_MOESM1_ESM.docx]

**
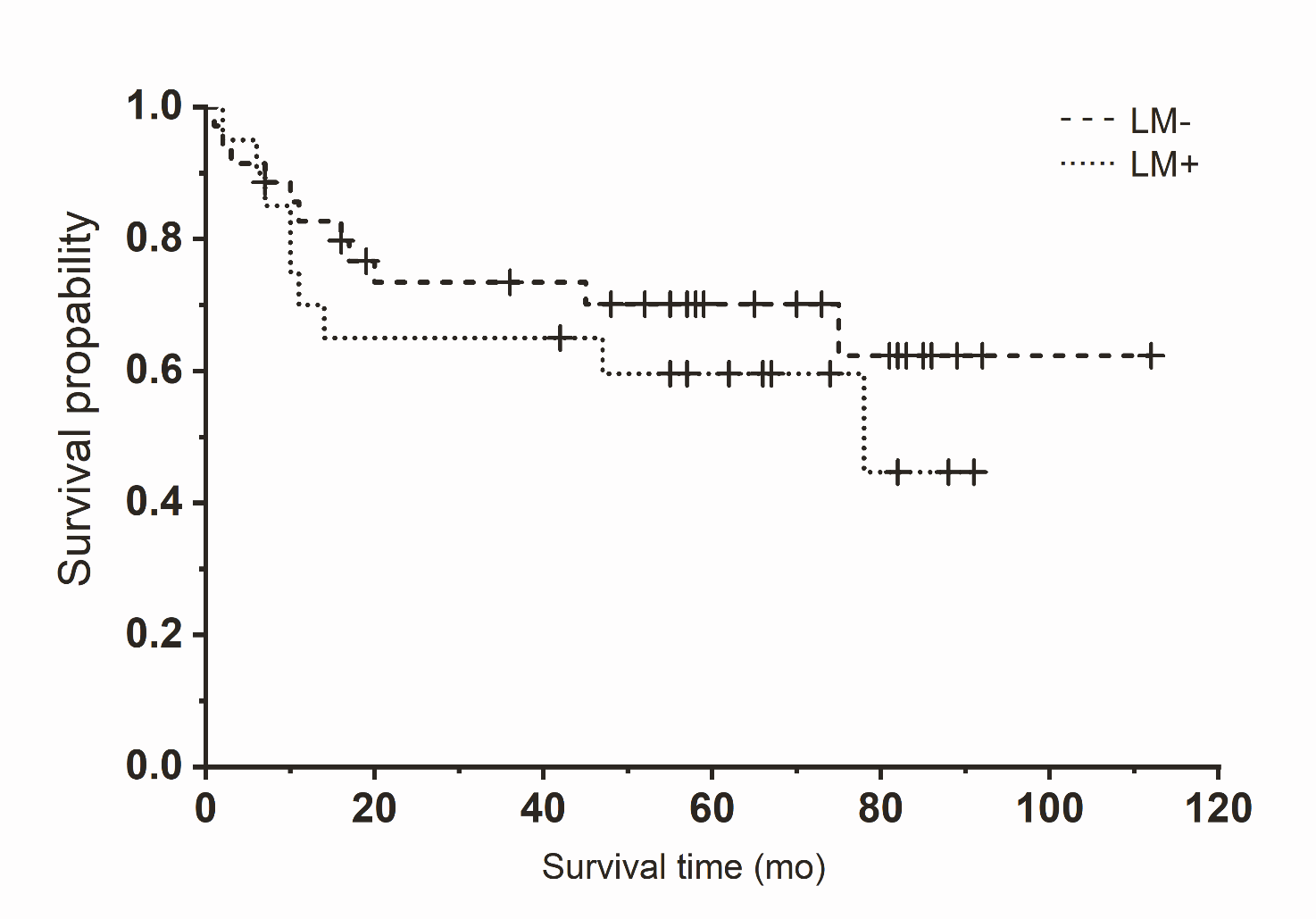
Supplementary Fig. 1. Estimated survival of oral squamous cell carcinoma patients with and without lymphatic mimicry.** The LM^+^ OSCC patients (n = 57) had shorter overall survival time (58 months) compared to patients without these structures (LM^-^, 80 months) albeit the difference was not statistically significant (P > 0.05). For patient data, the correlation between LM status and clinicopathological parameters was analysed with chi-square test. Survival was estimated with Kaplan-Meier analysis. LM, lymphatic mimicry; Mo, months; OSCC, oral squamous cell carcinoma. The investigators were blinded to the clinical data of the patients during the experiment and when assessing the outcome by using samples with coded labels with no reference to any respective group.

**Supplementary Table 1.** Comparison of clinicopathological characteristics of oral squamous cell carcinoma patients with (+) and without (-) immunodetection of lymphatic mimicry (LM).

|  | **LM status (total n = 57)** | |  |
| --- | --- | --- | --- |
| *Characteristics* | *LM- (n)* | *LM+ (n)* | *P-value* |
| **Age (years)** |  |  |  |
| <45y | 0 | 3 | 0.016 |
| >45y | 37 | 17 |  |
| **Sex** |  |  |  |
| Male | 22 | 13 | 0.682 |
| Female | 15 | 7 |  |
| **cT** |  |  |  |
| cT1-2 | 32 | 14 | 0.077 |
| cT3-4 | 4 | 6 |  |
| **pT** |  |  |  |
| pT1-2 | 30 | 15 | 0.422 |
| pT3-4 | 3 | 3 |  |
| **cN** |  |  |  |
| 0 | 30 | 14 | 0.176 |
| 1 | 4 | 5 |  |
| **pN** |  |  |  |
| 0 | 11 | 8 | 0.525 |
| 1 | 5 | 4 |  |
| 2 | 0 | 1 |  |
| **Grade** |  |  |  |
| 1 | 6 | 8 | 0.217 |
| 2 | 20 | 9 |  |
| 3 | 7 | 3 |  |
| **Recurrence** |  |  |  |
| No | 23 | 14 | 0.443 |
| Yes | 11 | 4 |  |

**
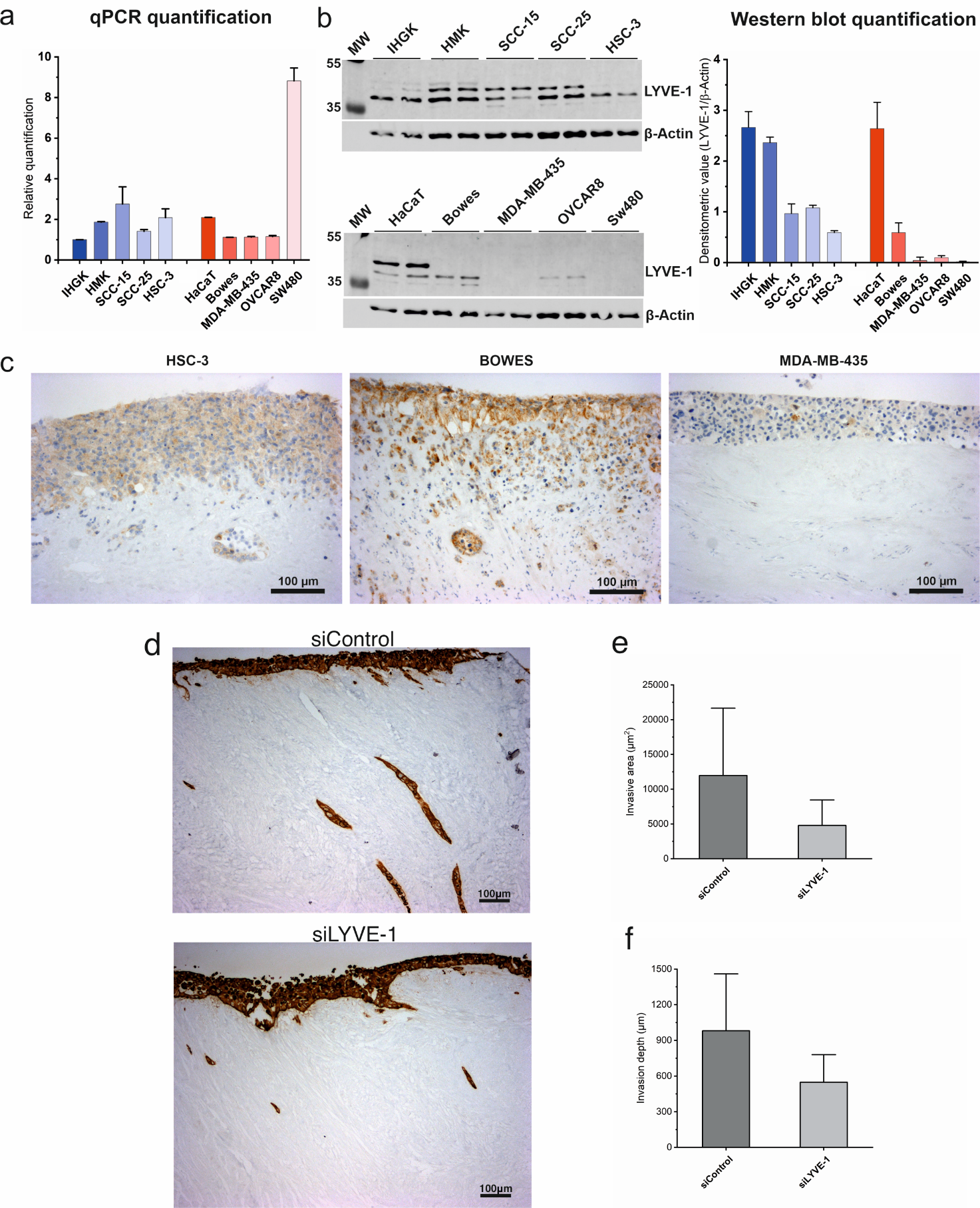
Supplementary Fig. 2. Expression of LYVE-1 in multiple different cell lines. (a-b)** The qPCR and Western blotting analyses showed that various keratinocytes and cancer cell lines differentially express LYVE-1 in vitro. **(c)** Organotypic myoma 3D model showed that LYVE-1 immunoreactivity was retained in the cancer cell lines. **The pro-invasion potential of LYVE-1 in 3D in vitro model (d)** Representative figure of the 3D Organotypic Myoma model with siControl and siLYVE-1 HSC-3 cells. CK staining, 10x magnification. **(e-f)** The siLYVE-1 HSC-3 cells had less invasion area and invasion depth in myoma organotypic model compared with the siControl, however, the difference was not statistically significant. Scale bar 100 µm. Data are presented as means ± standard deviations.

**
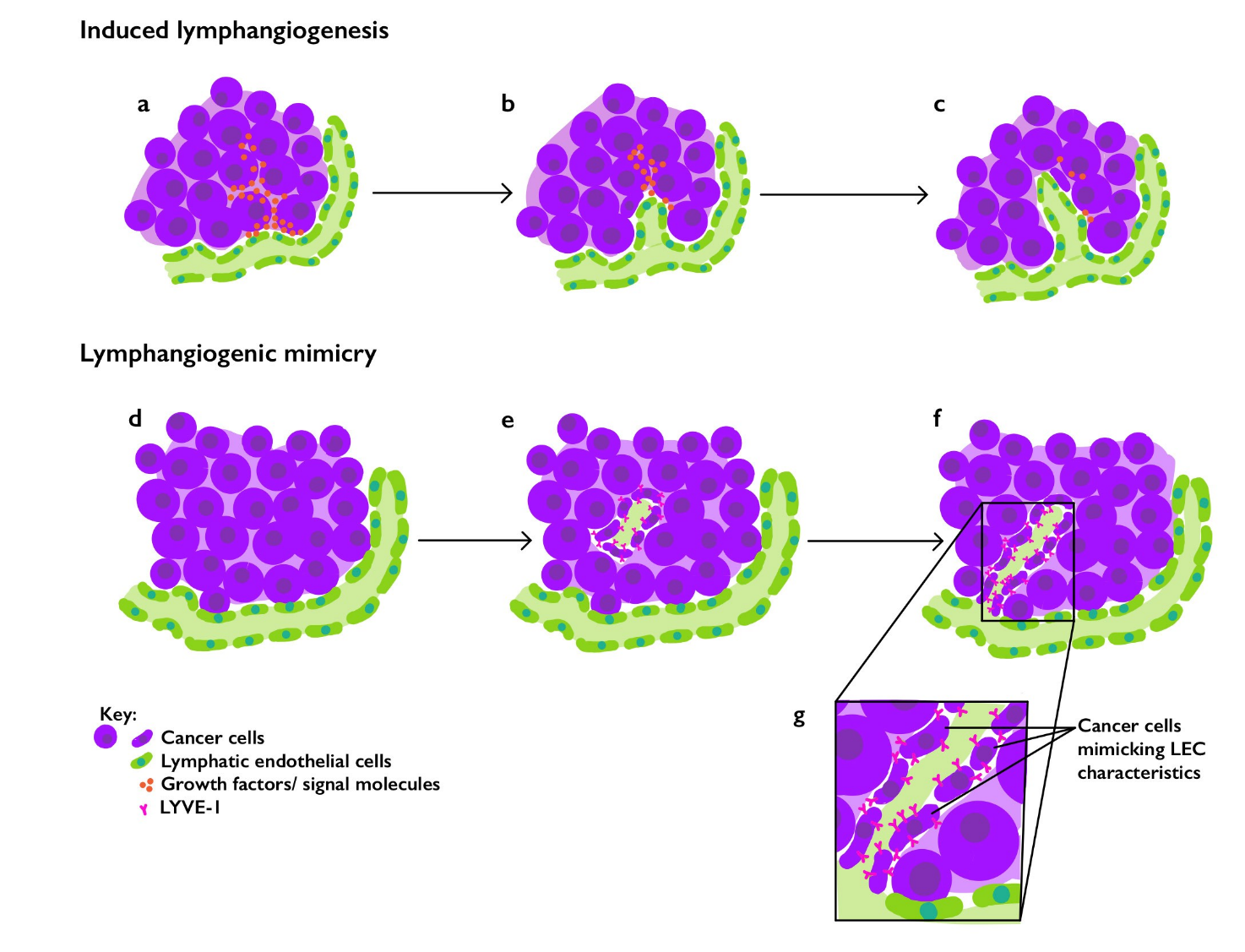
Supplementary Fig. 3.** **A comparison between an induced lymphangiogenesis and lymphatic mimicry (LM) in oral carcinogenesis.** In the induced lymphangiogenesis: **(a)** Tumour cells secrete lymphangiogenic factors; **(b)** the lymphangiogenic factors induce the budding of pre-existing lymph vessels; **(c)** new lymphatic vessels lined with lymphatic endothelial cells (LEC) are formed. In LM: **(d)** Aggressive cancer cells in a densely crowded and hypoxic tumour take advantage of their phenotype plasticity; **(e)** tumour cells attain LEC-like phenotype and initiate lymph vessel-like structures; **(f)** these *de novo* channels may connect to an existing lymphatic vessels and enhance tumour growth and metastasis. **(g)** LM structures are lined by tumour cells that exhibit the LEC-specific marker (i.e. LYVE-1).

**Supplementary materials and methods**

Cell culture and sample collection conditions of this study were as follows: The high-metastatic human HSC-3 cell line (JCRB Cell Bank; Osaka National Institute of Health Sciences, Osaka, Japan), and the low-metastatic SCC-25 cell line (ATCC, Rockville, MD, USA) were cultured in Dulbecco’s Modified Eagle medium (DMEM) with Nutrient Mixture F-12, 10% heat inactivated foetal bovine serum (FBS), 100 U/ml penicillin, 100 μg/ml streptomycin, 50 μg/ml ascorbic acid, 250 ng/ml fungizone, and 0.4 ng/ml hydrocortisone (Life Technologies, Grand Island, NY, USA). The HSC-3 and SCC-25 cell lines were recently authenticated with an overall identity estimate of 100% (Technology Centre, Institute for Molecular Medicine Finland FIMM, University of Helsinki). Primary LEC cell line (a kind gift from Prof. Lauri Eklund, University of Oulu) was cultured in Endothelial Cell Media MV2 (C22200, PromoCell) for DDPCR analysis. All cells were cultured in a humidified +37°C and 5% CO_2_ air atmosphere. Cells were detached using trypsin-EDTA (Sigma-Aldrich), counted and plated in cell culture well-plates (Corning, New York, NY, USA). Cancer cell lines SCC-15 (ATCC CRL-1623) and MDA-MB-435 were cultured as similar to HSC-3 and SCC-25 cells. Cancer cell line SW480 (gifted by Dr. Tatiana Rinaldi) was cultured in Leibovitz's L12 supplemented with 100 U/ml penicillin, 100 μg/ml streptomycin, 250 ng/ml fungizone and 10% heat-inactivated fetal bovine serum. Cancer cell line OVCAR8 (gifted by prof. Kaisa Lehti, University of Helsinki, Finland) was cultured RPMI 1640 supplemented with 1x Insulin-Transferrin-Selenium mix, 2mM L-glutamine, 100 U/ml penicillin, 100 μg/ml streptomycin, 250 ng/ml fungizone and 10% heat-inactivated fetal bovine serum. Skin HaCat cells and cancer cell line BOWES (ATCC CRL-9607) were cultured in DMEM supplemented with 100 U/ml penicillin, 100 μg/ml streptomycin, 50 μg/ml ascorbic acid, 250 ng/ml fungizone, 1 mmol/L sodium pyruvate and 10% heat-inactivated fetal bovine serum. Oral keratinocyte cell lines HMK and IHGK Keratinocyte were cultured in SFM with supplements (Human Recombinant Epidermal Growth Factor & Bovine Pituitary Extract), 100 U/ml penicillin, 100 μg/ml streptomycin, 250 ng/ml fungizone and 100µM calcium chloride. All cell lines were cultured in humidified +37°C 5% CO_2_ atmosphere, apart from BOWES cell line, which was cultured in normal air at +37°C. For RNA extraction, 1.5 x 10^5^ cells (cancer cell lines) or 3 x 10^5^ (keratinocyte cell lines) were plated in duplicates on 6-well plates (Corning). For protein extraction, 4 x 10^5^ cells (cancer cell lines) or 8 x 10^5^ (keratinocyte cell lines) were plated in duplicates in T25 bottles (Corning). After 3-day incubation, the cells were washed with cold PBS and subjected for RNA extraction performed with RNeasy mini kit (Qiagen) or for protein extraction as described in methods section. RNA quantity was measured with NanoDrop 2000 (Thermo Fisher Scientific) and cDNA was generated with RevertAid First Strand cDNA synthesis kit (Thermo Fisher Scientific). By using the primers described for ddPCR as well as FastStart Universal SYBR Green Master Mix (Roche), the LYVE-1 gene expression was quantified with qPCR in Rotor-Gene 3000 machine (Qiagen) and normalized to the corresponding housekeeping gene GAPDH values. GAPDH primers were ordered from Sigma-Aldrich and consisted of the following sequences: forward 5’-CACCAACTGCTTAGCACCC (cat. no. 130207620-001), reverse 5’-GCAGGGATGATGTTCTGGA (cat. no. 130209321-030). Orthotopic mouse model of OSCC: tumour cell suspensions in cold serum-free DMEM (Sigma-Aldrich) were mixed with cold Matrigel (1 : 1) to a final concentration of 8 × 10^6^ cells/ml. The orthotopic human OSCC xenografts were established by injecting cell suspension (25 µl containing 2 × 10^5^ cells) of parental and control HSC-3 cell suspension into the lateral tongue of seven-week-old BALB/c nude male mice (Charles River, Germany). Control HSC-3 were transduced with a non-coding control sequence (Amsbio, Abingdon, UK). Mice were then weighed and monitored regularly until euthanasia was administered using CO2 at day 13 (post-injection) or if they lost more than 10% of their body weight. The tongues and draining lymph nodes were removed and fixed in 4% formalin, imbedded in paraffin and were cut to 4 µm-thick sections. The investigators were blinded to the animal group allocation during the experiment and when assessing the outcome by using samples with coded labels with no reference to any respective group. The experiments followed the ARRIVE guidelines and were conducted in accordance with the European Convention for the Protection of Vertebrate Animals for Experimental and Other Scientific Purposes´ guidelines on accommodation and care of animals. Myoma assays were performed as described by Nurmenniemi et al.^20^. For invasion assays, cells were cultured on top of myoma tissue for 5 days due to the transient nature of the silencing effect. The samples were fixed in 4% PFA and embedded in paraffin. Five µm sections were cut, deparaffinised and rehydrated (Tissue‐Tek^®^DRS™2000). Antigen retrieval was performed in Tris‐EDTA buffer (pH 9) microwaving in Milestone T/T Mega Multifunctional Microwave Histoprocessor (Shelton). After cooling, the slides were washed with PBS–Tween^®^ 20 (0.01%). Dako REAL™ peroxidase blocking solution (Dako, 10 min RT) was used for blocking endogenous peroxidase activity. After washing as described before, sections were incubated for 1 hour at RT with polyclonal rabbit antihuman LYVE-1 antibody (1:6000; HPA042953, Sigma‐Aldrich); or for 30 min at RT with monoclonal mouse antihuman pan-cytokeratin antibody (1:200; M3515, Dako), in Dako REAL™ Diluent (Dako). After another washing, the sections were incubated with Dako HRP rabbit/mouse horseradish peroxidase (Dako REAL™ Envision™) for 30 min. Counterstaining was done with Mayer's haematoxylin. The staining was visualized under Leica DM4000B microscope using 10x or 20x objectives and imaged with Leica LCF320 camera using Leica Application Suite software. The image analyses were performed with Fiji platform^1^.

**Supplementary Videos 1.** The HSC-3 cells are forming well-defined interconnected vessel-like network on Matrigel. Images were taken using the IncuCyte S3™ Live-Cell Imaging System (24 hours).

**Supplementary Videos 2.** The siControl HSC-3 cells are still forming consistent interconnected vessel-like network on Matrigel. Images were taken using the IncuCyte S3™ Live-Cell Imaging System (24 hours).

**Supplementary Videos 3.** The siLYVE-1 HSC-3 cells lost their capability to form any consistent vessel-like network on Matrigel. Images were taken using the IncuCyte S3™ Live-Cell Imaging System (24 hours).

**Reference:**

1. Schindelin, J. et al. Fiji: an open-source platform for biological-image analysis. *Nat. Methods*. **28**, 676-682 (2012).
